# A soft, transparent, freely accessible cranial window for chronic imaging and electrophysiology

**DOI:** 10.1038/srep27818

**Published:** 2016-06-10

**Authors:** Chaejeong Heo, Hyejin Park, Yong-Tae Kim, Eunha Baeg, Yong Ho Kim, Seong-Gi Kim, Minah Suh

**Affiliations:** 1 Center for Neuroscience Imaging Research (CNIR), Institute for Basic Science (IBS), Suwon 16419, Republic of Korea; 2 Department of Biological Science, Sungkyunkwan University, Suwon 16419, Republic of Korea; 3 SKKU Advanced Institute of Nanotechnology (SAINT), Sungkyunkwan University, Suwon 16419, Republic of Korea; 4 Department of Biomedical Engineering, Sungkyunkwan University, Suwon 16419, Republic of Korea

## Abstract

Chronic *in vivo* imaging and electrophysiology are important for better understanding of neural functions and circuits. We introduce the new cranial window using soft, penetrable, elastic, and transparent, silicone-based polydimethylsiloxane (PDMS) as a substitute for the skull and dura in both rats and mice. The PDMS can be readily tailored to any size and shape to cover large brain area. Clear and healthy cortical vasculatures were observed up to 15 weeks post-implantation. Real-time hemodynamic responses were successfully monitored during sensory stimulation. Furthermore, the PDMS window allowed for easy insertion of microelectrodes and micropipettes into the cortical tissue for electrophysiological recording and chemical injection at any location without causing any fluid leakage. Longitudinal two-photon microscopic imaging of Cx3Cr1^+/− GFP^ transgenic mice was comparable with imaging via a conventional glass-type cranial window, even immediately following direct intracortical injection. This cranial window will facilitate direct probing and mapping for long-term brain studies.

To better comprehend neural function and connectivity in a living brain, it is desirable to have a large-scale cranial window that can maintain normal brain conditions for as long as possible. Furthermore, to better utilize the recent advances in neuroscience techniques to study the wide brain network^1^,^2^,^3^,^4^,^5^, an ideal cranial window should have the following properties: 1) high optical clarity and a wide-field of view for longitudinal morphological and functional imaging and optogenetic stimulation, 2) simple fabrication process for any size and design of window, and 3) easy accessibility for the introduction of pharmacological drugs, dyes, and viruses at desired locations as well as for electrophysiological recording to be performed at any position within the cranial window.

Cranial windows in rodents require open-skull or thinned-skull procedures^6^,^7^,^8^,^9^. The well-known open-skull cranial window techniques involve a full craniotomy, in which an exposed area of the brain is sealed with a cover glass without filler material^6^,^8^ or filled with either agarose^6^ or silicone^10^. In rats, the dura mater is removed because of its thick and opaque properties, but dural regrowth hinders optical transparency in longitudinal imaging^6^. Furthermore, full duratomy can easily initiate an inflammatory cascade, thereby preventing for the success of a long-term large cranial window in rats.

In mice, thinned-skull cranial window procedures have been used to minimize inflammation of the cortical area^8^,^11^,^12^,^13^, but, as is the case with rats, the skull needs to be repeatedly thinned due to regrowth^12^, thus reducing the utility of this approach for longitudinal studies. To reduce the effects of bone regrowth and perform longitudinal imaging studies, a cover glass is mounted onto a thinned area with cyanoacrylate cement^7^. These cranial window techniques facilitate relatively large-area live-brain imaging with high clarity using optical approaches such as two-photon (2P) microscopy^14^,^15^. However, the cover glass used in both open- and thinned-skull cranial window techniques is impenetrable. Recently, partial solutions to these problems have been proposed, such as using removable cranial windows to inhibit dura regrowth effectively^16^, drilling a small access port to the side of a cover glass to inject calcium dye through the port^17^, or attaching a microfluidic channel under a cover glass^18^. However, these methods are not without drawbacks.

Furthermore, an inherent problem in using glass as a cover window is its rigidity, which limits its ability to cover large brain areas with curvature. The diameter of a typical cranial window is approximately 3 mm for open-skull windows and approximately 1 mm for thinned-skull windows. As the window size increases, the possibility of the cover glass applying unwanted pressure on the cortical tissue arises, especially in rats, in which it may disrupt cerebrospinal fluid regulation and intracranial pressure levels. Thus, *in vivo* chronic optical imaging studies in rodents using current cranial window techniques are mostly confined to small areas due to the difficulties of maintaining stable physiological conditions for large brain areas. Biocompatibility of PDMS has been previously shown in the studies, where PDMS was used as an artificial dura within a glass-type cranial chamber in long-term primate visual studies^19^,^20^. In primate studies, large area duratomy is needed to expose the visual cortex for clear cortical imaging. However, it is very challenging to sustain brain health for large duratomy in monkeys. PDMS-like silicone materials are suitable as a transparent artificial dura within the cranial window with glass cover in monkeys. To overcome the limitations of conventional cranial window approaches, we propose a novel and simple cranial window technique using a brain cover based on soft, flexible, elastic, clear, and biocompatible PDMS.

## Results

### Highly flexible and simple cranial window for chronic functional *in vivo* imaging

**Figure 1:**
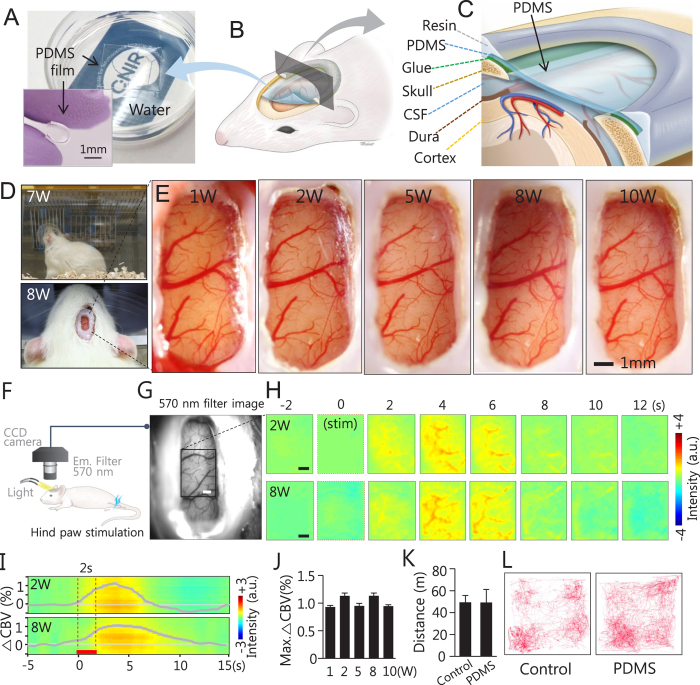
PDMS chronic cranial window development and installation and cortical vascular structural and functional observations during long-term studies. (**A**) A hydrophobic and transparent square-type PDMS pad is floating on water and the clearly visible logo is placed under the culture dish. An insert image shows the bending feature of PDMS. (**B,C**) Schematics of the cranial window of rodents with flexible PDMS covering. (**D**) An animal with PDMS window behaves naturally in its home-cage 7 weeks post-implantation (top, and Supplementary Video S1) and no sign of inflammation around the implantation is visible 8 weeks post-implantation (bottom). (**E**) Magnified cortical images of the PDMS cranial window at 1, 2, 5, 8 and 10 weeks post-implantation. Despite temporal sequences clear vasculature is evident in all images. (**F–J**) Optical recording of intrinsic signal (ORIS) via PDMS window. (**F**) ORIS was performed with total haemoglobin weighted 570 nm wavelength during electrical stimulation (1 mA, 3 Hz, 2 s) of the left hind paw in three animals. (**G**) One example shows a clear brain surface image obtained using ORIS (scale bar: 1 mm). (**H**) A robust CBV change was observed after stimulation. The time course of peak intensity changes was obtained from the active region of 2.96 × 2.96 mm^2^ (Black box in G, scale bar: 1 mm). Group-averaged time course of CBV change at 8-weeks post-implantation (n = 3, mean ± SEM) is plotted in (**I**) and the average maximum CBV changes (mean ± SEM) at 1, 2, 5, 8 and 10 weeks post-implantation (n = 3 for each group) are shown in (**J**). Red bar in (**I**): 2 s stimulation period. In comparison with CBV changes at 1w, no significant differences were found in CBV changes at 2w (p = 0.70), 5w (p = 0.87), 8w (p = 0.67), and 10w (p = 0.79) with two-tailed Student’s t-test. (**K,L**) Home cage activity of a rat with a PDMS cranial window was studied and compared with control rat. (**K**) No significant moving distance difference was found for 30 minutes activity in each group (n = 3, p = 0.99, two-tailed Student’s t-test). The graph shows in mean ± SD. A single animal’s navigation pattern from each group is shown in (**L**).

A silicone-based organic polymer film comprising PDMS is highly flexible and can float on water due to its light weight and hydrophobic nature (Fig. 1A and Supplementary Fig. S1). PDMS can be easily fabricated to have any desired thickness and appropriate softness by mixing the elastomer base and curing agent in a 10:1 ratio in a sterilized culture dish and by curing 1–2 h at 80 °C. This flexible material can be tailored to any size or shape, which allows the cover to be well fitted onto the curved cortex, thereby, creating a large cranial window (Fig. 1B). It is well known that PDMS is highly transparent and has equal absorbance of light for wavelengths between 300 nm and 700 nm (Supplementary Fig. S2). Moreover, we cultured cells on PDMS and found that the viability exceeded 95%, which suggests that PDMS does not cause cellular toxicity (Supplementary Fig. S3A,B). These confirm that PDMS is a soft, flexible, transparent, and biocompatible material. The schematic diagram demonstrates how the soft and flexible cover material can be used as a cranial window in rats (Fig. 1B,C). We performed open-skull craniotomy of the rat with a large area covering almost one hemisphere between the bregma and lamda (Supplementary Fig. S4). In a simple surgical procedure description (see the online Methods section for details), following craniotomy and duratomy, an approximately 0.3-mm thick PDMS pad with a size of 5 × 11 mm^2^ was used to cover the exposed curved cortical tissue (approximately 4 × 9 mm^2^) and was carefully glued to the skull around the exposed cortex (green line in Fig. 1C between the PDMS and skull) using a cyanoacrylate adhesive. Then, the boundary of the PDMS cranial window was sealed with a dental resin. Over time, the biocompatibility of the material kept the skin surrounding the window healthy, and there were no noticeable signs of infection or discomfort when the individual animal was housed in its home-cages. Examples of animal conditions at 7–8 weeks post-implantation are shown in Fig. 1D & Supplementary Video S1. These results were also supported by previous reports, where biocompatible PDMS was used as an artificial dura under a conventional cranial chamber^19^,^20^ and as a substrate in the implantable neural-muscle interface of rat spinal cord injury^21^.

The ability to capture *in vivo* images repeatedly is a crucial factor in evaluating the success of a cranial window. In an open-skull cranial window with a glass cover in rats, a duratomy is required for better visibility due to inflammatory reactions as well as dural regrowth^6^,^11^,^12^,^16^, which makes it difficult to maintain an optically clear window for an extended period of time. To examine the condition of an open-skull chronic window with PDMS, 13 rats subjected to a duratomy procedure were studied for up to 10 weeks after the cranial window implantation. Healthy cortical vasculature underneath the cranial window was constantly visualized over a large area of the rat brain (Fig. 1E in one rat & Supplementary Fig. S5 in other rats). Furthermore, neither cell and tissue adhesion nor noticeable bone regrowth on the PDMS were apparent during the long-term observations, possibly due to the hydrophobicity of PDMS (Supplementary Fig. S1A,B). In cell culture experiments, neuronal cells rarely attached to PDMS, silicone, or agar though they did have a high affinity towards glass (Supplementary Fig. S3B).

Additionally, for acute *in vivo* experiments (Supplementary Fig. S6), PDMS can be used as a skull/dura-like substitute by directly covering exposed cortical regions^22^, which effectively prevents the cortical surface from drying and bulging when a large cortex area is opened for several hours. Similarly, flexible PDMS can be applied to a large curved thinned-skull area (Supplementary Fig. S7). In both thinned- and open-skull procedures, the PDMS cover reduces light scattering from an undulating surface and improves the visualization of cortical vessels and tissue. These properties of high transparency and flexibility make PDMS an excellent cranial window cover for large curved brain regions.

The cranial PDMS window allowed us to perform longitudinal wide-field-of-view *in vivo* imaging to determine functional changes in large cortical areas. Weekly optical recording of intrinsic signals (ORIS) (Fig. 1F–J) was performed during hindpaw electrical stimulation (2 section, 1 mA, 3 Hz) for up to 10 weeks after implantation of the cranial window. The experimental set-up for ORIS and hindpaw electrical stimulation is shown in Fig. 1F. A clear and large cortical surface image was obtained with a CCD-camera with a 570 nm filter (Fig. 1G). Robust hemodynamic responses upon stimulation were observed at 2 weeks and 8 weeks post-implantation (Fig. 1H,I), and the maximal changes in cerebral blood volume (CBV) remained similar over 10 weeks (Fig. 1J), thus indicating that the PDMS cranial window is an excellent material for long-term chronic imaging, even without the dura mater and skull. To support our *in vivo* data, histochemical analysis of microglial cells and astrocytes was performed after the ORIS experiments at 10-weeks post- implantation (Supplementary Fig. S8). We observed that microglial cells and astrocytes were similar in the cortical sections of the cranial window and intact skull. Similar functional and histochemical observations were made for all of the rats studied. The behavioral data obtained at 4-weeks post-implantation showed that the animals with PDMS cranial window exhibited no significant differences in moving distances (Fig. 1K) and patterns (Fig. 1L) compared to control animals.

### Easily accessible cranial window for penetration at multiple sites

**Figure 2:**
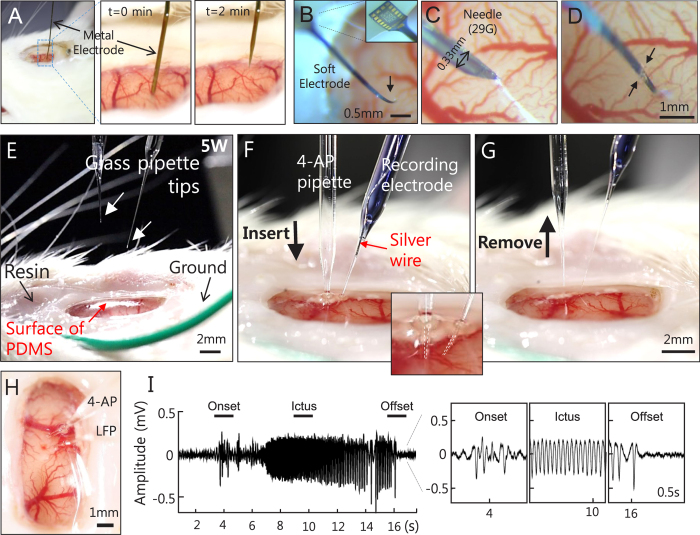
Accessing neural tissue directly through the PDMS cranial window over a rat brain. (**A**) A side view of the metal electrode penetration directly through the PDMS and its magnified images at time series. The injected electrode was slowly pulled out at a speed of 0.5 mm/min. No bleeding or CSF leakage was observed. (**B–D**) A soft and thin type of the multi-channel electrode alone could not penetrate (**B**) and bent upon contact with the PDMS surface (black arrow). (**C**) Penetration was aided by a sharp needle (29 gauge) and (**D**) the electrode was slid inside via a slit (black arrows). (**E–H**) Sequential images of the glass pipette placement and removal performed at 5 weeks post-cranial window implantation. Two glass pipettes were inserted into the brain tissue directly through the PDMS cover by avoiding major vasculature; one glass pipette (clear) was used to inject 700 nl of potassium blocker 4-aminopyridine (4-AP) at 15 mM concentration, to induce seizure activities, while the other pipette (blue with trypan blue dye for visualization) contained a silver wire filled with 0.9% NaCl, which was used to record local field potential (LFP). Tips of both pipettes were marked with arrowheads in E and outlined with dashed lines in an inserted, expanded image of (**F**). No noticeable leakage was detected after the pipettes were removed from the cranial window (**G,H**). To prevent the possible rupture of PDMS later, the insertion sites were glued with fast-acting adhesive cyanoacrylate (**H**). (**I**) Record electrophysiological signals induced by 4-AP. We observed a clear seizure onset, ictus, and offset. Expanded time courses shown at bottom right were obtained from three line-marked time periods.

To investigate brain function *in vivo*, it is often necessary to have direct access to the neural tissue. PDMS has superb flexibility (Fig. 1A, insert) and, exceed 200% elongation at break (Supplementary Table S1), which enables repeated neural tissue penetration without jeopardizing the integrity of the cranial window. In Fig. 2A, side and magnified views demonstrate penetration by a generic metal electrode and its smooth exit through the PDMS cranial window (Fig. 2A). After the inserted electrode was pulled out slowly at 0.5 mm/min, no bleeding or CSF leakage was noticeable. In the case of recording electrodes, which are fragile and lack stiffness (Fig. 2B), a small guiding incision (Fig. 2C) was made with a 29 -gauge syringe needle prior to insertion of the electrode (Fig. 2D). Again, these procedures did not induce any CSF leakage (Supplementary Video S2) owing to the high elasticity and viscosity of PDMS. The low wettability of PDMS, which has a high water contact angle (99.9°) and, consequently, a high hydrophobicity, also has a role in leakage prevention (Supplementary Fig. S1). If the object being inserted is too large (greater than 20 gauge, 0.51 mm) the PDMS may rupture, but this can be easily remedied by applying fast-setting cyanoacrylate glue over the rupture (Supplementary Fig. S9).

Multi-site penetrations were easily performed by sliding two glass pipettes, one each for drug injection and electrophysiological recording through the flexible PDMS material and, avoiding large vessels. Their tips were positioned approximately 1 mm below the cortical surface for injection and approximately 0.5 mm below the surface for recording (Fig. 2H). 4-aminopyridine (4-AP), a fast potassium channel blocker, was injected, and the spontaneous seizures induced by 4-AP were recorded. Well-defined seizure onset, ictus, and offset were observed from local field potential (LFP) recordings obtained by the recoding electrode (Fig. 2I). Although no CSF leakage was found after removal of the electrodes, clear fast-setting cyanoacrylate glue was applied over the penetration sites to prevent any potential ruptures of the PDMS (Supplementary Fig. S9A). After five weeks, the injection sites maintained their high visibility and integrity (Supplementary Fig. S9B). These results demonstrate that the PDMS cranial window can be used to directly access neural tissues at any site multiple times.

### Longitudinal cellular resolution two-photon imaging

**Figure 3:**
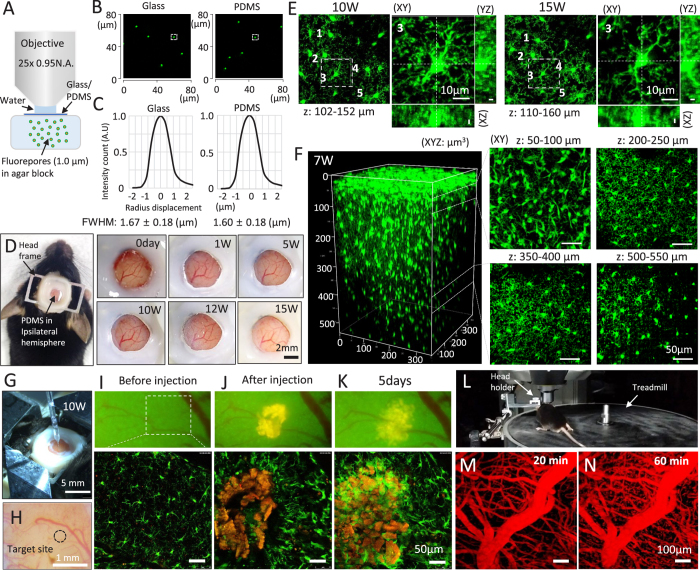
Two-photon (2P) *in vivo* microscopy and microglial immunohistochemistry of a Cx3Cr1^GFP+/−^ Tg mouse with a PDMS chronic cranial window. (**A–C**) Measurements of 2p confocal microscopic signal intensities through a glass and PDMS cover for 1 μm fluorophore micro-beads which were embedded in a 1% agarose block (**A**). No differences were found in 2p images (**B**), and in the averaged signal intensity profile judged by FWHM (**C**). (**D**) A picture of the mouse with a cranial window and a head frame. Images were captured from a single mouse at longitudinal time points from day 0 to 15 weeks post-surgery. (**E**) Maximum projection 2p images at 10 and 15 weeks of the same regions denoted by the numbers from 1 to 5 on each microglia around 100 μm in z-depth (Z-thickness: 50 μm). Magnified image of cell number 3 shows ramified morphological branches with high resolution and XY, XZ, and YZ images are well represented through PDMS window. All scale bars: 10 μm. (**F**) 2P imaging for microglial cells was possible up to 600 μm in Z-depth (XYZ). (**D**) XY images are shown magnified cells at different depths. (**G**) The Cy3-labeled hydrogel dye injection through PDMS mouse window at 10 weeks post-implantation by using conventional glass micropipette. A glass micropipette with Cy3-labeled hydrogel dye (140 nl) is directly inserted into the target site (H, a dotted circle) with a nanoliter-injector. (**I**) The cortex of the target site was shown in by the florescence transmitted light image (upper panel). Microglia image (lower panel) of the white dotted box in upper panel (**I**). (**J**) After injection of the Cy3-labeled dye, Cy3 fluorescence signal is clearly shown at the target position under florescence transmitted light microscope and 2p imaging, upper and lower panels, respectively. Longitudinal 2p imaging of microglia cells are observed before and after the injection at ~70 μm in Z-depth. Whole z-depth images of hydrogel and microglia are shown in Supplementary Fig. S12B. (**L**) Awake mouse is walking over a treadmill during 2p imaging. (**M,N**) Cortical surface vasculatures of behaving mouse are visualized after rhodamine-dextran injection up to 60 minutes.

Large open-skull PDMS cranial windows in transgenic mice are useful for free accessing brain tissue and performing 2p imaging of deep cortical layers for an extended period of time. The resolution of the confocal microscopic signal through PDMS was tested with 1.0 μm fluorescent micro-spheres (Fig. 3A,B) and the full width of half maximum (FWHM) of signal intensity was calculated from the normalized intensity profile in Fig. 3C. The FWHM of the signal obtained via PDMS (~1.60 μm ± 0.18 μm, mean ± SD, n = 15) was not significantly different (p = 0.99, two-tailed Student’s t-test) from that of the signal via glass (~1.67 μm ± 0.18 μm, mean ± SD, n = 15).

For *in vivo* 2p imaging, a large PDMS cranial window of approximately 5 mm in diameter was implanted over the intact dura in Cx3Cr1^GFP+/−^ Tg mice expressing green fluorescent protein in the microglia, and a square-type head frame was used to secure the head of the mice onto a stage-frame (Fig. 3D). To determine the effects of the cranial window on the health of the mice, behavioral test and wide-field optical imaging were performed for up to 25 weeks after implantation. No unusual behavior was observed in any of the 18 mice (see Supplementary Video S3, a representative mouse at 25 weeks post-implantation). Time-lapse cranial window images were captured from the day of the surgery up to 15 weeks post-surgery (Fig. 3D), which showed that, similarly to the observations made in rats with cranial windows, normal healthy vasculature was clearly maintained. Overall, mice with the open-skull PDMS cranial windows remained healthy up to 25 weeks post-implantation. However, bone and dura regrowth was evident around approximately 35 weeks post-surgery.

Longitudinal 2p microscopy imaging was used to track microglia activities and cortical microvascular structures. We identified same microglial cells at 10-weeks and 15-weeks post-surgery (Fig. 3E) and observed their detailed ramified morphologies in XY, XZ, and YZ axis with high resolution. Taking advantage of the clarity of the PDMS cranial window, we conducted 2P imaging up to a depth of 600 μm and visualized microglia cells (Fig. 3F). At a cortical layer depth of 50–100 μm, perivascular microglial cells were distributed alongside large cortical vessels, whereas deeper layers showed ramified microglia, indicative of normal physiological conditions in the brain without any invasion by foreign materials or cell apoptosis^23^. To visualize the cortical vessels by 2P imaging, a solution of dextran-conjugated fluorescence dye was injected into the retro-orbital venous sinus (Supplementary Fig. S10). Both vasculature and microglia were clearly imaged with 2P imaging at 7 and 15 weeks post-implantation. Real-time blood flow was also observed using an *in vivo* fluorescence microscope (Supplementary Video S4). To confirm our 2P data, the microglia and astrocyte distributions in the cortex were evaluated by immunohistochemistry at 1, 3, and 10 weeks after implantation (Supplementary Fig. S11). The microglia cell density in the ipsilateral chronic window side of the cortex increased at 1 week after implantation compared with the contralateral normal side but returned to normal at 3 weeks (Supplementary Fig. S11A). Similarly, at 1 week post-surgery, the astrocytes were over-activated near the surface on the ipsilateral side of the cortex compared with the contralateral side. After 3 weeks, the astrocyte distribution in the ipsilateral cortex appeared homogeneous and was similar to the contralateral side (Supplementary Fig. S11B). In addition, vessel staining was prominent on the top layer of the ipsilateral side of the mouse brain at 1 week post-surgery but decreased at 3 and 10 weeks post-surgery (Supplementary Fig. S11C).

We further studied the longitudinal interactions of microglia to a foreign protein in Cx3Cr1^GFP+/−^ Tg mice with PDMS cranial window (Fig. 3G–M). Cy3-labeled protein hydrogel dye, which can prolong the fluorescence signal in tissue, was used to test the *in vivo* dynamic reaction of microglia. The small volume of Cy3 dye (140 nl) was directly injected into the cortex through PDMS cranial window by a glass micropipette and nanoliter-injector (Fig. 3G). The injection site was randomly selected to avoid blood vessels (Fig. 3H). Immediately after removal of the glass micropipette, the sign of injection was barely noticed under the light surgical microscope (maximum magnification upto 60x), and continued invisible at 5-days post-injection, even without bond or glue application (Supplementary Fig.S12A). At 15 minutes after the injection, 2p confocal microscopic images can profile how the injected Cy3 hydrogel dye (red) interacts with microglia (green) in the cortical tissue and show Cy3 dye distributed across through 100 μm in z-depth (Supplementary Fig. S12B). The injection site could be confirmed through 2p image on the PDMS cover (Supplementary Fig. S12B, white arrows in z-depth at 1 μm). Moreover, the longitudinal 2p imaging up to 20 days post-injection allows chronic investigation of the dynamic responses of microglia to the injected Cy3 dye (Fig. 3I–K, lower panels). Up to the first week, the microglia showed fast clustering towards the Cy3 dye, whereas between 1 week and 3 weeks, the peri-neighboring microglia were returned to normal condition (Fig. 3I–K, lower panels) as the Cy3 signal became diffuse and weak (Fig. 3I–K, upper panels). This strongly suggests that our soft and transparent PDMS window offers excellent opportunities to study long-term brain responses to foreign dyes or chemicals, which involve *in vivo* injection directly via the PDMS window. In addition, we kept animals awake for *in vivo* imaging. An awake mouse with a PDMS window was walking on a treadmill for 1 hour during a 2p imaging session. As shown in Fig. 3L, the head-fix frame of the PDMS window was secured onto a bar attached to the side of the treadmill and was placed under the 2p microscope. We imaged cerebral vasculature (Fig. 3M,N) every 20 minutes after rhodamine-dextran (70,000 molecular weight, 1.5 μl per 1g body weight) injection. There was a slow and continuous leakage of rhodamine-dextran into a tissue area for over 60 minutes. A video-clip of an awake 2p imaging session is shown in Supplementary Video S5. This demonstrates our PDMS window can be successfully sustained during *in vivo* imaging for both anesthetized and awake conditions.

Recently, in the systems neuroscience research field, neural stimulation in one region and detection in the target region have become essential to the study of neuronal connectivity. Neural activities transmitted to the projecting region can be evoked by electrical, optogenetic, or chemical stimulations and 2p imaging, or conventional electrophysiological recording can be performed at the target region^2^,^3^,^4^,^5^. Additionally, injecting viral vectors, drugs, or chemicals into the tissue at a single or multiple time points have become important for elucidating neural network connectivity and the underlying functions^17^,^24^,^25^,^26^,^27^. With our open-skull wide-view PDMS cranial window, we were was able to i) maintain animals at healthy conditions up to 15 weeks after the surgical implantation, ii) insert multiple microelectrodes and micropipettes for electrophysiological recording and chemical injection at any location, and iii) obtain longitudinal functional imaging with high clarity and 2p imaging with deep tissue penetration. These observations meet the highly ideal criteria for using a chronic cranial window to study system neuroscience.

High optical homogeneity and clarity in PDMS films can be obtained by constant thickness and degassing air bubbles. PDMS is an appropriate optical material for applications such as micro-lenses with a refractive index of 1.48; it also has minimum light absorption in the visible light spectrum range (Supplementary Fig. S2) and extremely low autofluorescence^28^,^29^. These optical properties persist up to a thickness of approximately 0.42 mm and PDMS can be used optimally as a light-guiding immersion material up to this thickness^29^. Of the various PDMS films we tested, films with a thickness of 0.25 to 0.35 mm showed optimal physical characteristics and stability for window and proved easier to handle than thinner films. For instance, when the film thickness was below 0.15 mm, the film was prone to rolling up upon contact with the forceps and to rupturing upon penetration by an electrode. Biocompatibility and feasibility of PDMS for long-term implantation were tested in previous studies, where PDMS was used as an artificial dura under a glass-cover within a cranial chamber in primates^19^,^20^. In our study, we vastly simplified this scheme by combining the artificial dura, glass-cover, and cranial chamber into an integrated system solely consisting of a biocompatible PDMS cover with an appropriate thickness without any need for additional cover materials or chambers for rodents.

A chronic wide-view cranial window can be easily made with PDMS. The sizes of our chronic PDMS cranial windows were up to 36 mm^2^ in rats and 18 mm^2^ in mice, which are much larger than the previously reported chronic cranial window sizes^6^,^7^,^8^,^11^,^13^,^16^. To the best of our knowledge, no successful chronic rat windows lasting more than one week exist due to the inflammatory reactions caused by removal of the full dura mater as well as dural regrowth^6^,^30^. In mice, the cranial window size is confined to a small area ranging from hundreds of microns to a maximum of 5 mm in diameter^6^,^8^,^16^. In the case of thinned–skull cranial windows for imaging spine dynamics, the typical size of the cranial window is several hundred microns (approximately 0.2 mm) in diameter^11^,^13^. Recently, an advanced method using a polished and reinforced thinned skull has reported a window 1–2 mm wide^7^. When a small area in mice must be imaged repeatedly, the use of a glass cranial window is efficient. In addition, a hard-type window (a non-elastic material cover) can disrupt the local CSF flow because the hard cover could generate pressure down to the cortex, thus resulting in a narrow space between the cover and cortical tissue. Unlike hard-type windows, PDMS, owing to its expandability, allows sufficient space for the normal CSF flow conditions to be maintained. Therefore, we suggest that this feature makes our soft cranial window an ideal option for long-term wide-view brain monitoring.

The softness of PDMS may allow it to be more accommodating to brain motion than hard cover materials and may not interfere with CSF flow. Consequently, dynamic cortical vessel pulsations were detected through PDMS open-skull cranial windows, and similar results are expected for thinned-skull cranial windows (Supplementary Videos S2 and S4 in rat and mouse, respectively). As you can see in video the cortical pulsation by respiratory is found in the rat with PDMS cover (Supplementary Video S2). It is insignificant in the mouse case (Supplementary Video S4). All 2p images shown in Fig. 3 were obtained with raw image data-set without post-signal processing indicating pulsation artifacts were minimal. Only at the surface of the cortex, motion artifacts were observed. Imaging conditions were stable in the deeper layers. To get the high-resolution image for rat case, motion artifacts in time-lapse images can be mitigated by applying standard post-hoc image analysis. Conventionally, to stabilize brain motion, clear and non-bio agarose is used to fill the space between the brain tissue and cover glass^6^,^14^,^18^.

The high viscoelasticity of PDMS allows for the insertion of microelectrodes and micropipettes without causing any fluid leakage, thus conferring a major advantage over other cranial window materials. Furthermore, the hydrophobicity of PDMS may further reduce the chance of solution leakage through the small insertion slit and keep the cranial window transparent for the entire duration of the experiment, even in chronic rat cranial windows without dura mater (Fig. 2 and Supplementary Video S2). In a previous study, Roome and Kuhn^17^ developed a cover glass with a small PDMS port to insert a glass pipette for the injection of a calcium dye for *in vivo* calcium imaging. Although the PDMS port in their study was shown to be resealable after injections, our large PDMS window offers the additional advantage of multiple penetrations at any desirable location with multiple times.

Thus, PDMS is ideal for the construction of chronic cranial windows allowing insertion of multiple electrodes at any location. We expect that the large-scale soft cranial window techniques presented here, when combined with other modern neurotechnologies, such as optogenetics, will contribute to the elucidation of brain function and circuitry.

## Methods

### PDMS film preparation

Polydimethylsiloxane (PDMS, SYLGARD 184, Dow Corning, USA) film was prepared by mixing the base elastomer and curing agent in a 10:1 (v/v) ratio, degassing completely within a vacuum desiccator (−0.1 MPa, Pfeiffer vacuum pump, Germany), and then solidifying within a sterilized cell culture dish (diameter 100 mm) in an oven incubator (80 °C, 1–2 h). The PDMS film was sterilized by autoclaving and UV exposure. It could be cut into any desired shape, and the dimensions and thickness were regulated according to the volume in the dish. For example, 4 ml of PDMS solution in the culture dish (diameter 100 mm) formed a film that was 330 (±30) μm thick. We used 250–350 μm thick PDMS covers for the mouse cranial windows, which were thinner than those used for rats because the area to be covered and the skull thickness of mice are much smaller. It should be noted that the PDMS film should have a constant thickness and be solid, without any micro-bubbles. These characteristics can be obtained by degassing completely in the vacuum chamber and by ensuring that the oven is flat when incubating the PDMS solution. Tapping the side edges of the culture dish from all directions several times or using a spin coater can also help evenly spread the PDMS solution in the culture dish.

### Transparency and hydrophobicity measurements

An image of the materials (empty polystyrene culture dish), PET, Agarose, Silicone, PDMS, Glass) in a 12-well culture plate is shown in Supplementary Fig. S1A. The thicknesses were as follows: culture dish (polystyrene) = 1.3 mm, glass = 0.15 mm, agarose = described 1 mm, PDMS = described 0.3 mm, PET = described 0.05 mm, and silicone = described 0.4 mm. The transparency of each material was measured by analyzing light absorption on a microplate reader (Synergy HT, BioTek, USA). The microplate reader scanned the absorbance of each well over 300–700 nm at 10 nm increments. We measured and averaged the absorbance values from multiple points in each well. These values were normalized against the value of an empty well and shown in mean value. Low absorbency (A) means a high transparency (T) in terms of the Beer-Lambert equation, T = 10^−A^. The absorption curve is shown in Supplementary Fig. S2. The contact angle was measured using water to evaluate the surface wettability (PHX300, Surface Electro Optics, South Korea). A low angle (<90°) corresponds to high wettability, indicating hydrophilicity, whereas a high angle (>90°) corresponds to low wettability, indicating hydrophobicity (a water drop image and contact angle are shown in Supplementary Fig. S1).

### *In vitro* cell study

SHSY5Y neuroblastoma cells (ATCC, USA) which are growing as a mixture of floating and adherent cell types were cultured in DMEM (Gibco BRL, USA) media containing 10% heat-inactivated fetal bovine serum (Gibco BRL, USA) and 1% penicillin/streptomycin (Invitrogen, USA) in a T75 flask and were subcultured every 3 days. The cells were routinely washed with phosphate-buffered saline (PBS, Welgene, South Korea) and detached using cell dissociation solution (Sigma-Aldrich, USA). To observe cell growth on various materials, sterilized materials were placed into each well of a 12-well plate (Nunc, Thermo Scientific, USA), and 1 × 10^4 ^cells in 2 ml of media were loaded per well. The plate was incubated at 37 °C, 5% CO_2_ for 24 h. Cells were then collected and counted using a haemocytometer counting chamber (Marienfeld, Germany) after mixing with trypan blue solution (Sigma-Aldrich, USA) at a 1:1 ratio. For cell viability, the ratio of live cell (%) was calculated by dividing total cell number by live cell number. The statistical test was performed by two-tailed student’s t-test for each group against control (*p < 0.05, n = 5 for empty, glass, agar, PET, and n = 8 for PDMS, Silicone. n = number of well). The cells were imaged via phase contrast optical microscopy (AF6000B, Leica, Germany).

### Animal preparation and behavioral testing

Male Sprague-Dawley rats (Orient Bio Inc., South Korea) weighing 280–320 g and male and female C57BL6 and Cx3Cr1^GFP+/−^ mice (Jackson Laboratory, USA) were used. This study was reviewed and approved by the Institutional Animal Care and Use Committee (IACUC) of Sungkyunkwan University School of Medicine (SUSM). All animal experiments including surgical methods were performed in accordance with the approved guidelines by IACUC of SUSM. The SUSM facility is accredited by the Association for Assessment and Accreditation of Laboratory Animal Care International (AAALAC International) and abides by the Institute of Laboratory Animal Resources (ILAR) guide. The animals were housed in an SPF facility with 60% humidity at 23 °C under a 12 h light/dark cycle and were fed food and water *ad libitum*. The rats were randomly chosen at 2, 4, or 7-weeks post-PDMS implantation and marked with black ink on their backside. The rats were placed in their home cages with bedding but without cage lids and their behavior was recorded with a camera for 30 min using a video tracking system (EthoVision XT, Noldus, Netherlands).

### Cranial window implantation in rats and mice

The animals were anaesthetized in an induction chamber with 3.0% isoflurane (Hana Pharm., South Korea). They were supplied with pure O_2_ during anaesthesia (VetEquip) for 1–2 min, and then maintained with 2.0% of isoflurane during surgery for the cranial window implantation and 1.3% isoflurane during imaging. The level of anaesthesia was assessed through toe pinching during surgery and experimentation. The animals’ body temperatures were maintained at 36.5–37.5 °C using a heating pad connected to a controller (DC temperature controller, FHC, USA), and their eyes were covered with eye protecting gel (Solcorin, Solco Basie Ltd). During experimentation, heart rate (mouse = 300–450 beat/min, rat = 300–400 beat/min) and O_2_ saturation (SpO_2_; mouse >95%, rat >99%) were monitored using a pulse oximeter (Rat: Nonin Medical, USA and mouse: Kent Scientific, USA). Their heads were firmly fixed with ear bars in a stereotaxic frame (David Kopf Instruments, USA). An incision was carefully made over the skin of the right hemisphere (Fig. 2A for rats). The remaining epidermis and debris were removed with isopropyl alcohol and betadine liquid swabs. *For the rat surgeries*, a craniotomy (3 mm above bregma and 6 mm below bregma with a width of 4 mm) was performed through careful drilling using a dental drill (MICROTORQUE II, NJ, USA). After craniotomy, the dura mater was quickly removed with fine forceps. The exposed brain tissue was subsequently covered with PDMS (size = 6 mm × 11 mm, thickness = approximately 0.4 mm), and the edge of the PDMS was immediately glued onto the skull using cyanoacrylate glue (Loctite, USA). When air was trapped between the cortical tissue and PDMS, the air bubbles were removed by pushing ACSF solution into the space between the cortical tissue and the PDMS using a syringe. *For the mouse surgeries*, we implanted a PDMS cranial window and used a head-fixing stereotaxic frame for two-photon microscopy. To secure the head-fixing frame onto the skull, one microscrew (1.6 mm, Plastics one, USA) was embedded in the olfactory bulb site and another microscrew was embedded in the contralateral hemisphere. We then made an outline of a circle with a 4–5 mm inner diameter over the somatosensory area, between the bregma and lambda, and the head-fixing square frame (size = 12 mm ×19 mm ×1 mm, Narishige, Japan) was secured to the sketched circle using cyanoacrylate glue. Immediately, we carefully cut along the circle line to remove the skull using a dental drill. The sterilized PDMS was prepared and soaked in saline solution. Subsequently, a sterilized PDMS film (circle with a diameter of 6 mm and thickness of 0.25 mm–0.35 mm) was applied to fully cover the exposed circle-shaped brain tissue, and the boundary of the PDMS was glued onto the skull. We waited approximately 10 min to allow for drying. After tight sealing of the PDMS onto the skull using cyanoacrylate glue in both rats and mice, a dental resin (OA2, Dentist Inc., South Korea) was applied along the edges of the PDMS and exposed to UV light for 10 s to ensure permanent implantation within the skull (steps showed in Fig. 2). The remaining incision area was sutured and disinfected with antiseptic liquid. The animals were administered Meloxicam (1 mg/kg, Boehringer Ingelheim, Germany) and Baytril (5 mg/kg, Bayer, Germany) via subcutaneous injection and returned to their cages. Subsequently, Tylenol (1 ml/10 ml, Janssen, USA) was administered to ameliorate any potential pain, and Septrin (1 mg/ml, Samil Pharmaceutical Company, South Korea) was provided in the drinking water to relieve inflammation. A video showing a freely moving mouse with a PDMS cranial window is shown in the supplementary Video S3 (25 weeks). Before each imaging session, 70% Ethanol or distilled water was used for PDMS cleaning. Transparent PDMS surface becomes opaque by Acetone. In some cases, air bubbles can be found inside PDMS window (between the PDMS and cortex). These bubbles usually disappear naturally in 2–5 weeks post-surgery. In very rare cases, it takes longer than 5 weeks for the bubbles to disappear. So far, we have conducted PDMS cranial window surgeries on over 130 animals including rats (n = ~30) and mice (n = ~100). Out of over 130 animals with the implantation, only one male mouse broke the whole window frame with dental resin 15 months post-implantation. Furthermore, less than 10% of the rats and 5% of the mice showed inflammation inside the window. The percentage of successful animal preparations for imaging is at least as high, if not higher, than the glass window preparation method.

### Electrophysiology for 4-AP induction

We introduced two glass micropipettes into the brain tissue to record spontaneous neuronal activity directly via the PDMS cranial window. The glass micropipettes were made using a micropipette puller (P1000, Sutter instrument, USA). One electrode filled with 0.9% NaCl was used for extracellular recording of the local field potential (LFP), and the other electrode was filled with the drug 4-AP, a potassium channel blocker, for inducing ictal events. The electrodes were controlled and advanced through the PDMS cranial window under close visual control using a 3-axis micromanipulator (Narishige, Japan). The animal’s head was firmly secured to a stereotaxic frame (David Kopf Instruments, USA). Because a full duratomy had been previously performed (the time point was 5 weeks post-surgery), the glass pipette electrodes smoothly penetrated the PDMS and slid well into the recording and injection sites with the manipulators. The tips for injecting and recording were positioned at 1 and 0.5 mm below the surface of cortex, respectively. The injection of 4-AP (15 mM) was controlled with a microinjector (Nanoject II, Drummond, USA) and 700 nl were administered at a time. The ground wire was connected to a microscrew placed in the skull above the olfactory cortex. The electrophysiological experiments were performed on an anti-vibration table, within a Faraday cage under dark conditions. The LFP signal was amplified and filtered between 0.1 and 1000 Hz (ISO-DAM8A; World Precision Instruments, Inc.) and then digitized using a CED Power 1401 (Cambridge Electronic Design, Cambridge, UK).

### ORIS (optical recording of intrinsic signal) imaging and hindpaw stimulation

The animals were anaesthetized using 3% isoflurane inhalation in the induction chamber and maintained with 1.3% isoflurane on the stereotaxic frame for optical recording of the intrinsic signal (ORIS). For each animal, the head was fixed in a stereotaxic frame using ear bars and body temperature was maintained using a temperature controller (DC temperature controller, FHC, USA). The cranial window was evenly illuminated using an LED lamp (Leica, Germany). The left hindpaw of the animal was then electrically stimulated by inserting 30-gauge needle electrodes (Grass Products, WI, USA), connected to a pulse isolator (ISO-Flexor, A.M.P.I., Israel) and pulse stimulator (Master 9, A.M.P.I., Israel), under the skin between the second and third toes. Electrical stimulation (monophasic, 1 mA, 3 Hz) was administered for 2–5 s after the onset of ORIS. The intrinsic optical signal from the cortex was obtained using an optical imaging system (Imager 3001-Celox, Optical-Imaging, Inc., Jerusalem, Israel) equipped with a CCD camera (PhotonFocus AG, Switzerland) using a 570-nm band-pass filter to detect both oxygenated and deoxygenated hemoglobin, i.e., analogs to total hemoglobin and cerebral blood volume^31^,^32^. The overall recording time was 20 s, and the images were acquired with 200 frames (10 frames per second). Image acquisition was repeated 10 times (10 = number of stimulation) for each rat, and the results were averaged in every pixel. The images acquired from the ORIS system were analyzed using a custom MATLAB program (The MathWorks, Inc., USA). The frames captured 5 s before stimulation were considered as the baseline signal. The images were divided by the baseline images. A low-pass filter was applied for the pre-processing of the raw images to remove respiration artefacts. To calculate relative changes in the cerebral blood volume, we selected regions of interest (ROIs, 300 × 300 pixels, 1 pixel = 9.8 μm), and the pixel values within the ROIs were counted and plotted over time.

### Histological analysis

The entire brain was carefully extracted from animals after PDMS cranial window implantation using a microsurgical procedure following perfusion with an isotonic sodium chloride solution and a 4% paraformaldehyde (PFA) solution in a clean fume hood. The brain was fixed with 4% PFA and sectioned using a cryotome (VT1200S, Leica, Germany) to produce 20- or 40-μm thick slices that were subsequently mounted onto slides. The brain tissues were incubated in methanol and PBS containing 0.5% Triton X-100 or 10% bovine serum albumin (BSA; Sigma-Aldrich, USA) and the appropriate primary antibodies for either 1–2 h at room temperature or overnight at 4 °C. The primary antibodies used were as follows: Iba-1 (1:500, Wako Chemicals, USA) as a microglia marker, GFAP (1:500, Millipore, Germany) as an astrocyte marker, and GluT1 (1:150, Abcam, UK) as a vessel marker. After washing with PBS, secondary Alexa Fluor 568 goat anti-mouse IgG and Alexa Fluor 488 goat anti-rabbit IgG (1:150, Molecular Probes, USA) were added to the cells for 2 h at RT in the dark. The tissues were then rinsed three times with PBS. For nuclear staining, DAPI (1:100 dilution, Molecular Probes, Thermo Fisher Scientific, USA) was added to the last wash with PBS buffer, followed by mounting. We examined the slides using a confocal microscope (TCS SP8, Leica Microsystems, Germany) with a white-light laser (wavelength 488 nm, 568 nm) for fluorescence and a 405 nm laser for DAPI imaging. The images were analyzed using the LAS X program (Leica Microsystems, Germany).

### Two-photon microscope imaging *in vivo*

We used a two-photon microscope (2P) system from Leica, which comprised an SPX-8 confocal microscope body (Leica Microsystems, Germany) and Chameleon Vision II multiphoton lasers (Coherent, USA). The experiments were conducted in a dark and non-vibrating environment. The mouse head was fixed with head-frame locking in stereotaxic bars on the stage, and the animal was maintained under anesthesia using a gas tube. The objective lens was either 25 × (0.95 N.A.) or 10 × (0.22 N.A.) and was immersed in water on the PDMS window in the mouse head (Fig. 3A). We measured the fluorescence intensity of microbeads with yellow-green (505/515) fluorescence in size of 1.0 μm polystyrene (Life Technologies, USA), which were embedded in a 1% (w/v) agarose block. The full width of half maximum (FWHM) was measured from the profile of normalized intensity of each image. For the *in vivo* GFP-microglia cell imaging and the red fluorescence Cy3-labeled protein hydrogel imaging, the 2P Laser was used at a wavelength of 920 nm, 3–30% laser power (~1850 mW in 920 nm), and approximately 30% detector gain. Scanning was advanced with 16 or 32 averages, Z-steps of 0.5 μm or 1 μm, a 1.25 zoom under the 25× objective lens (372 μm × 372 μm scanning area) and a 363.64 nm × 363.64 nm pixel size. Single scans with a depth of 300 μm were captured over nearly 40 min using a scan profile. The Z-stack image and cross-section image were generated by maximum projection in 3D using LAS X microscope software. To obtain Cy3-labeled protein hydrogel images, hydrogel was injected by glass micropipette. The injection volume was controlled using by nanoliter-injector (Nanoject II, Drummond, USA). After the removal of the micropipette we did not apply any treatments at the injection site on the PDMS. In the following 2p imaging session up to 20 days, water was dropped onto the PDMS window for a 25× water immersion lens as usual. In awake 2p imaging, the mouse was placed over the treadmill, while the head-holder was fixed in a bar attached to the side of the treadmill. The vasculature images were obtained, after injection of rhodamine-dextran (70000 molecular weight), with z-depth of 150 μm from the surface, a gap size of 2 μm, a line-average of 32 times, and a scan time of approximately 5 min.

## Additional Information

**How to cite this article**: Heo, C. *et al*. A soft, transparent, freely accessible cranial window for chronic imaging and electrophysiology. *Sci. Rep*. **6**, 27818; doi: 10.1038/srep27818 (2016).

## Supplementary Material

Supplementary Information

Supplementary Movie S1

Supplementary Movie S2

Supplementary Movie S3

Supplementary Movie S4

Supplementary Movie S5

## References

[b1] Insel T. R., Landis S. C. & Collins F. S. The NIH brain initiative. Science 340, 687–688 (2013). 23661744 10.1126/science.1239276 PMC5101945

[b2] Tye K. M. & Deisseroth K. Optogenetic investigation of neural circuits underlying brain disease in animal models. Nat. Rev. Neurosci. 13, 251–266 (2012). 22430017 10.1038/nrn3171 PMC6682316

[b3] Jennings J. H. & Stuber G. D. Tools for resolving functional activity and connectivity within intact neural circuits. Curr. Biol. 24, R41–R50 (2014). 24405680 10.1016/j.cub.2013.11.042 PMC4075962

[b4] Lim D. H., LeDue J., Mohajerani M. H., Vanni M. P. & Murphy T. H. Optogenetic approaches for functional mouse brain mapping. Front. Neurosci. 7, 1–15 (2013). 23596383 10.3389/fnins.2013.00054 PMC3622058

[b5] Buzsáki G. . Tools for Probing Local Circuits: High-Density Silicon Probes Combined with Optogenetics. Neuron 86, 92–105 (2015). 25856489 10.1016/j.neuron.2015.01.028 PMC4392339

[b6] Shih A. Y. . Two-photon microscopy as a tool to study blood flow and neurovascular coupling in the rodent brain. J. Cereb. Blood Flow Metab. 32, 1277–1309 (2012). 22293983 10.1038/jcbfm.2011.196 PMC3390800

[b7] Drew P. J. . Chronic optical access through a polished and reinforced thinned skull. Nat. Methods 7, 981–984 (2010). 20966916 10.1038/nmeth.1530 PMC3204312

[b8] Holtmaat A. . Long-term, high-resolution imaging in the mouse neocortex through a chronic cranial window. Nat. Protoc. 4, 1128–1144 (2009). 19617885 10.1038/nprot.2009.89 PMC3072839

[b9] Farrar M. J. . Chronic *in vivo* imaging in the mouse spinal cord using an implanted chamber. Nat. Methods 9, 297–302 (2012). 22266542 10.1038/nmeth.1856 PMC3429123

[b10] Dombeck D. & Tank D. Two-photon imaging of neural activity in awake mobile mice. Cold Spring Harb. Protoc, doi: 10.1101/pdb.top081810 (2014). 24987148

[b11] Xu H. T., Pan F., Yang G. & Gan W. B. Choice of cranial window type for *in vivo* imaging affects dendritic spine turnover in the cortex. Nat. Neurosci. 10, 549–551 (2007). 17417634 10.1038/nn1883

[b12] Yang G., Pan F., Parkhurst C. N., Grutzendler J. & Gan W.-B. Thinned-skull cranial window technique for long-term imaging of the cortex in live mice. Nat. Protoc. 5, 201–208 (2010). 20134419 10.1038/nprot.2009.222 PMC4690457

[b13] Yang G. . Sleep promotes branch-specific formation of dendritic spines after learning. Science 344, 1173–1178 (2014). 24904169 10.1126/science.1249098 PMC4447313

[b14] Helmchen F. & Denk W. Deep tissue two-photon microscopy. Nat. Methods 2, 932–940 (2005). 16299478 10.1038/nmeth818

[b15] Svoboda K. & Yasuda R. Principles of two-photon excitation microscopy and its applications to neuroscience. Neuron 50, 823–839 (2006). 16772166 10.1016/j.neuron.2006.05.019

[b16] Goldey G. J. . Removable cranial windows for long-term imaging in awake mice. Nat. Protoc. 9, 2515–2538 (2014). 25275789 10.1038/nprot.2014.165 PMC4442707

[b17] Roome C. J. & Kuhn B. Chronic cranial window with access port for repeated cellular manipulations, drug application, and electrophysiology. Front. Cell. Neurosci. 8, 379, doi: 10.3389/fncel.2014.00379 (2014). 25426027 PMC4227473

[b18] Takehara H. . Lab-on-a-brain: Implantable micro-optical fluidic devices for neural cell analysis *in vivo*. Sci. Rep. 4, 6721, doi: 10.1038/srep06721 (2014). 25335545 PMC4205880

[b19] Shtoyerman E., Arieli A., Slovin H., Vanzetta I. & Grinvald A. Long-term optical imaging and spectroscopy reveal mechanisms underlying the intrinsic signal and stability of cortical maps in V1 of behaving monkeys. J. Neurosci. 20, 8111–8121 (2000). 11050133 10.1523/JNEUROSCI.20-21-08111.2000 PMC6772749

[b20] Chen L. M. . A chamber and artificial dura method for long-term optical imaging in the monkey. J. Neurosci. methods 113, 41–49 (2002). 11741720 10.1016/s0165-0270(01)00475-7

[b21] Minev I. R. . Electronic dura mater for long-term multimodal neural interfaces. Science 347, 159–163 (2015). 25574019 10.1126/science.1260318

[b22] Heo C. . Flexible, transparent, and noncytotoxic graphene electric field stimulator for effective cerebral blood volume enhancement. ACS nano 7, 4869–4878 (2013). 23651168 10.1021/nn305884w

[b23] Davoust N., Vuaillat C., Androdias G. & Nataf S. From bone marrow to microglia: barriers and avenues. Trends Immunol. 29, 227–234 (2008). 18396103 10.1016/j.it.2008.01.010

[b24] Chen T. W. . Ultrasensitive fluorescent proteins for imaging neuronal activity. Nature 499, 295–300 (2013). 23868258 10.1038/nature12354 PMC3777791

[b25] Mohajerani M. H., McVea D. A., Fingas M. & Murphy T. H. Mirrored bilateral slow-wave cortical activity within local circuits revealed by fast bihemispheric voltage-sensitive dye imaging in anesthetized and awake mice. J. Neurosci. 30, 3745–3751 (2010). 20220008 10.1523/JNEUROSCI.6437-09.2010 PMC6632233

[b26] Ferezou I., Bolea S. & Petersen C. C. Visualizing the cortical representation of whisker touch: voltage-sensitive dye imaging in freely moving mice. Neuron 50, 617–629 (2006). 16701211 10.1016/j.neuron.2006.03.043

[b27] Stosiek C., Garaschuk O., Holthoff K. & Konnerth A. *In vivo* two-photon calcium imaging of neuronal networks. Proc. Natl. Acad. Sci. USA 100, 7319–7324 (2003). 12777621 10.1073/pnas.1232232100 PMC165873

[b28] Yu X., Wang Z. & Han Y. Microlenses fabricated by discontinuous dewetting and soft lithography. Microelectron. Eng. 85, 1878–1881 (2008).

[b29] Chang A.-T., Tseng S.-Y. & Hsu L. Optical guiding with cylindrical mirror system. Proc. of SPIE 7762, 77622T1–7, doi: 10.1117/12.861003 (2010).

[b30] Nishimura N., Rosidi N. L., Iadecola C. & Schaffer C. B. Limitations of collateral flow after occlusion of a single cortical penetrating arteriole. J. Cereb. Blood Flow Metab. 30, 1914–1927 (2010). 20842163 10.1038/jcbfm.2010.157 PMC3002886

[b31] Frostig R. D., Lieke E. E., Ts’o D. Y. & Grinvald A. Cortical functional architecture and local coupling between neuronal activity and the microcirculation revealed by *in vivo* high-resolution optical imaging of intrinsic signals. Proc. Natl. Acad. Sci. USA 87, 6082–6086 (1990). 2117272 10.1073/pnas.87.16.6082 PMC54476

[b32] Suh M., Bahar S., Mehta A. D. & Schwartz T. H. Blood volume and hemoglobin oxygenation response following electrical stimulation of human cortex. NeuroImage 31, 66–75 (2006). 16480899 10.1016/j.neuroimage.2005.11.030

