# Supplementary material for: A soft, transparent, freely accessible cranial window for chronic imaging and electrophysiology
Source: Sci Rep. 2016 Jun 10;6:27818. doi: 10.1038/srep27818 (PMC4901295; doi:10.1038/srep27818)
Supplement: Supplementary Information [file srep27818-s1.pdf]

Supplementary information

- ***Table (1), Figures (12), and Videos (5)***

A soft, transparent, freely accessible cranial window for chronic imaging and electrophysiology

Chaejeong Heo[1], Hyejin Park[1, 2], Yong-Tae Kim[3], Eunha Baeg[1], Yong Ho Kim[3,4], Seong-Gi Kim[1, 4], and Minah Suh[1, 4]*

Table1 | Flexible property of transparent materials

Material	Flexibility (elongation at break)
glass	-
Agarose (1%)	Poor (<10%)
PDMS	Very good (>200%)
PET	Good (100%< ~ <200%)
Silicone	Very good (>200%)

Thickness, glass: 0.15 mm, agarose: ~ 1 mm, PDMS: ~ 0.3 mm, PET: ~0.05 mm, silicone: ~ 0.4 mm.

Supplementary Table S1 Flexibility of glass, 1% agarose, PDMS, polyethylene terephthalate (PET), and silicone. Flexibility was determined by measuring a break point when the elongation was forced. PDMS and silicone have spring-like characteristics and are easily stretchable.

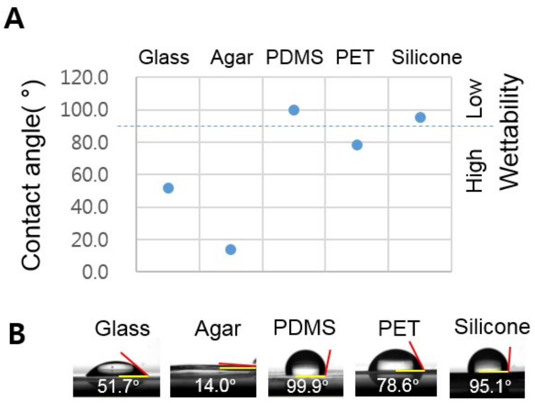

Supplementary Fig. S1 Physical hydrophobic property of glass, 1% agarose, PDMS, polyethylene terephthalate (PET), and silicone. (A) Contact angles and wettability of materials. (B) The contact angle represents an angle between a tangential line of liquid (red line) and solid surface where the liquid is located (yellow line) and is independent of drop volume. Wettability is high when the contact angle is close to zero and is low when the material is hydrophobic.

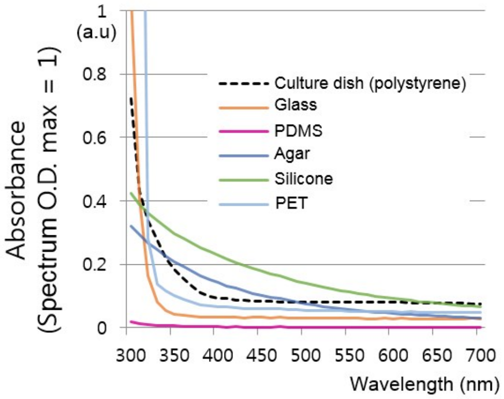

Supplementary Fig. S2 Light absorption curve of glass, agarose (1%), PDMS, polyethylene terephthalate (PET), and silicone. The light absorbance value of each material is plotted in every 1 nm over 300-700 nm wavelengths.

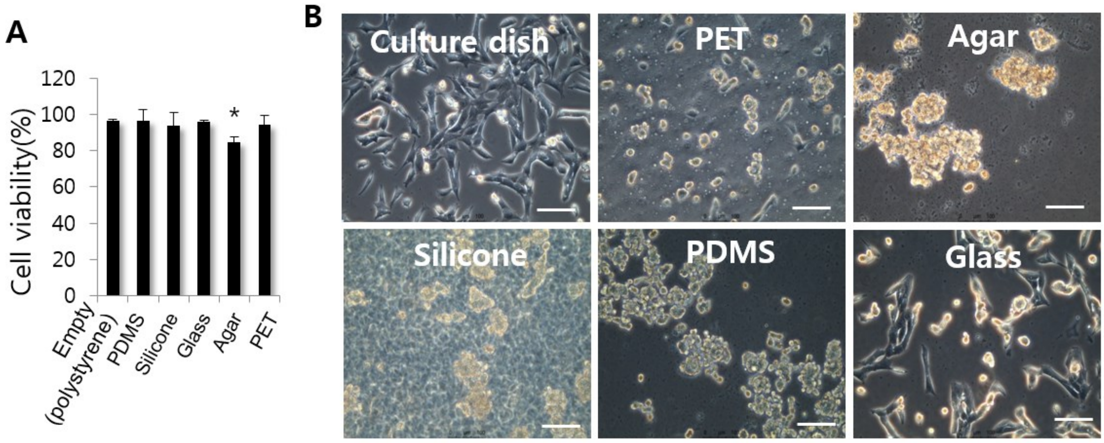

Supplementary Fig. S3 Biocompatibility of PDMS and other materials. SHSY5Y cells were cultured for 24 h over the materials, including PET, 1% agarose, silicone, PDMS, and glass, placed in the culture dish. (A) The cell viabilities of all tested materials except for agarose were high (>95%, error bar: mean ± SD), thus indicating high biocompatibility. (B) After 24 h of culture, the cells were attached to and grew on both the control case (culture dish) and the glass and were slightly attached to PET, whereas the cells were rarely attached to agarose (Agar), silicone, and PDMS (Scale bar: 100 μm). Blurry images of PET and silicone are due to the materials alone rather than to the presence of cells.

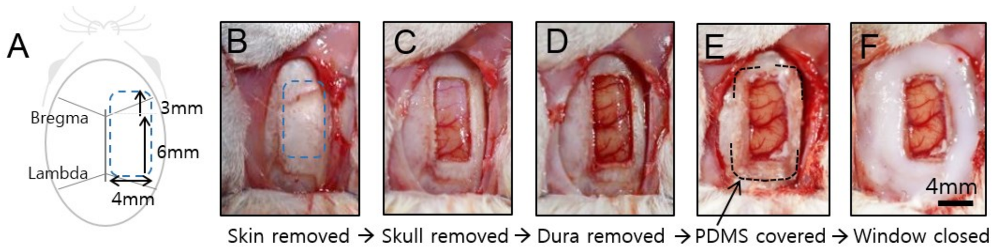

Supplementary Fig. S4 Overall surgical procedures for the chronic PDMS cranial window in a rat. (A) A schematic for a large rectangular (9 mm x 4 mm) skull craniotomy. (B-F) Visualization of each step of the cranial window surgical procedure. At each step, the skin (B), skull (C), and dura (D) were carefully removed. (E) A rectangular shape of the PDMS (black dot-lined) was placed over the exposed brain tissue area and glued over the intact skull. (F) A white-coloured resin was applied to secure the edge of the PDMS and cover the exposed skull area.

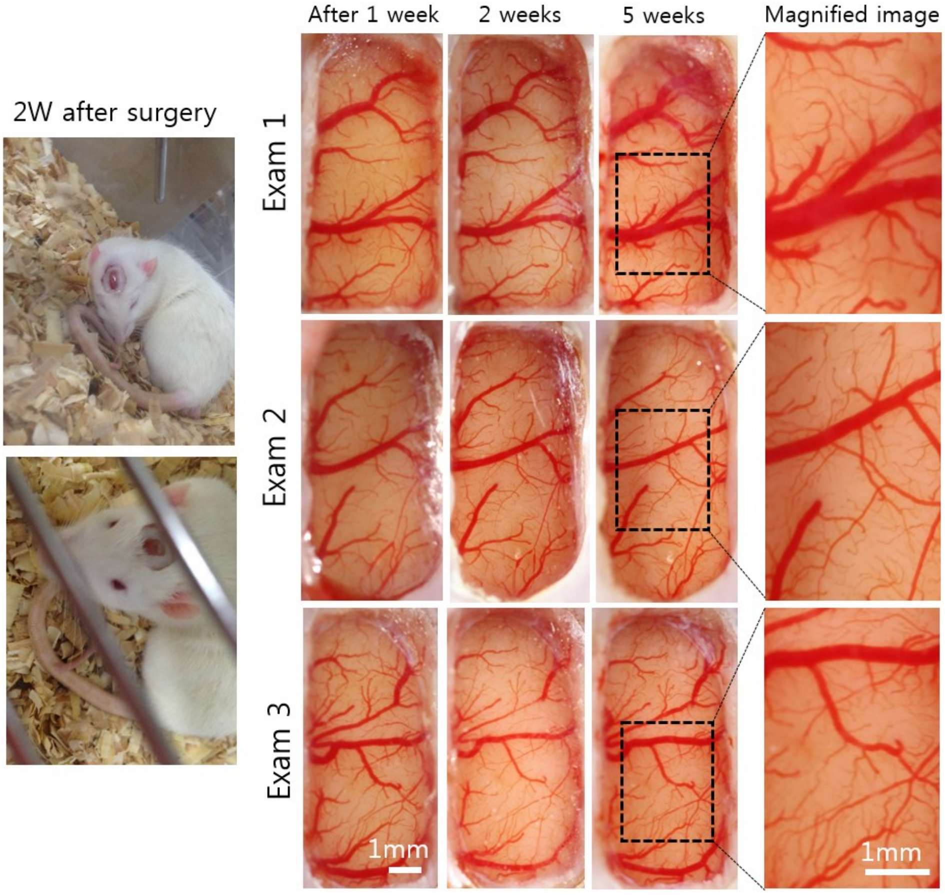

Supplementary Fig. S5 Examples of rat's cranial window conditions post-PDMS implantation. Generally after 2 weeks, animals exhibited normal activities in their own home cages. Three examples are shown the apparent cortical vasculature at 1, 2, and 5 weeks through a large scale PDMS cranial window. The window size was as described in Supplementary Fig. 4.

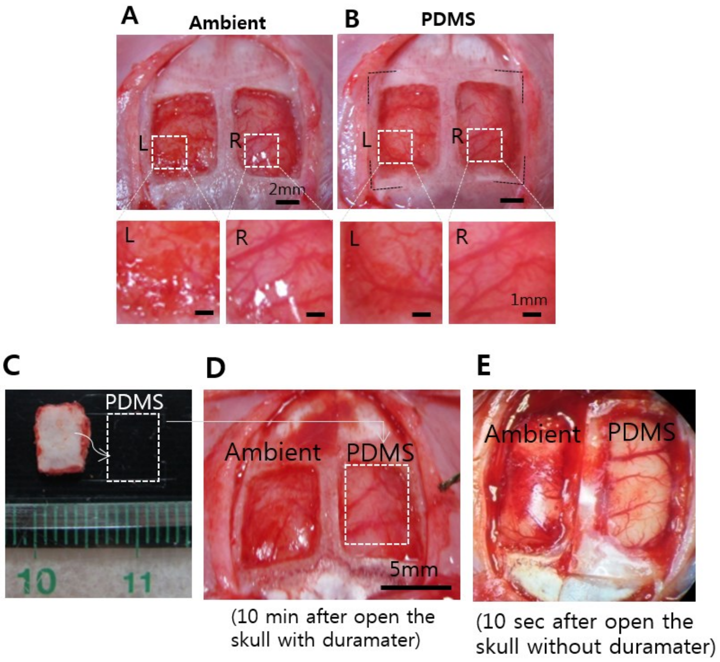

Supplementary Fig. S6 Effectiveness of PDMS in enhancing the optical (A-B) and physiological (C-E) condition in acute open skull experiments. (A-B) Images of a brain (A) without and (B) with a PDMS cover was captured after full craniotomy for both hemispheres of the rat brain. Brain with a PDMS cover exhibited less light scattering noise than brains without (B) a PDMS cover. (C) A comparison image of a square skull piece (right) after craniotomy and a tailored square PDMS (white-dotted box). (D-E) In general, when a bone-flap is removed from the region of interest during cranial window surgery, the brain tissue rapidly bulges through the open skull because of the greater intracranial brain pressure. When the duramater is maintained following skull opening, the exposed brain bulges after approximately 10 min (D, left). With full duratomy, brain tissue herniation can be observed even after 10 s (E, left). In contrast, the application of the PDMS cover prevents brain tissue herniation, even though the opened area is large (D and E, right), thus suggesting that the PDMS application might provide effective conditions for studying normal brain physiological function *in vivo*.

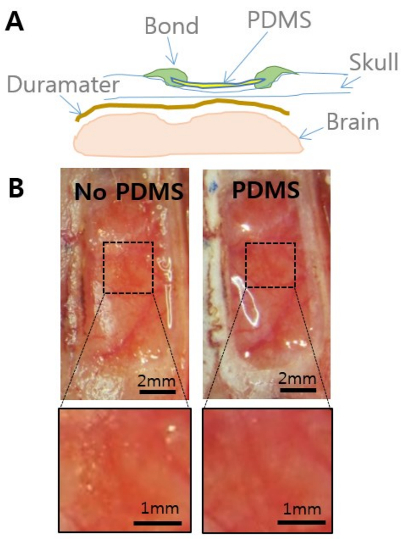

Supplementary Fig. S7 Effectiveness of PDMS for enhancing imaging quality in a thinned skull experiment. (A) A side-view schematic diagram of PDMS implantation over the thinned skull. (B) The large area of thinned-skull brain images without and with PDMS. An image with PDMS cover shows cortical vessels more clearly than an image without PDMS. The thickness of PDMS is ~150 μm.

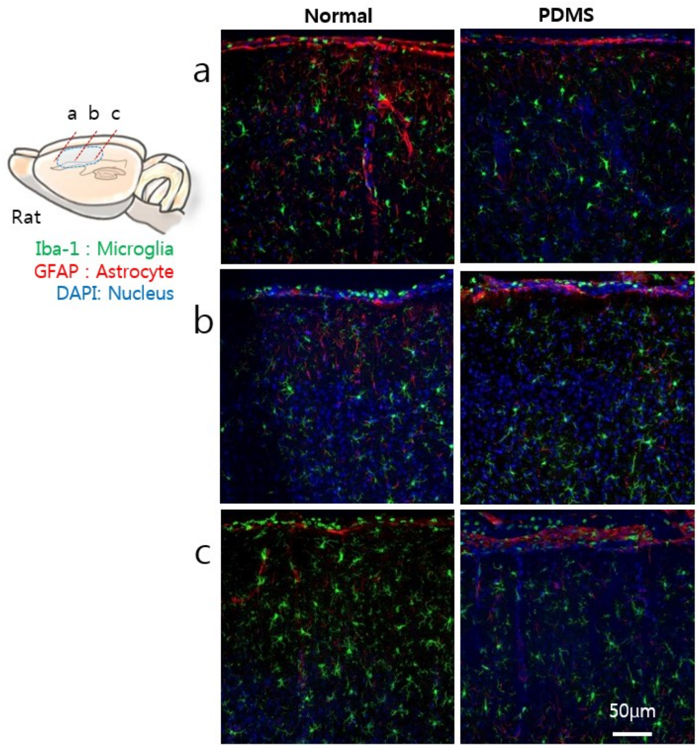

Supplementary Fig. S8 Immunohistochemical images for microglial cells (Iba1), astrocytes (GFAP) and nuclei (DAPI) of a rat with the PDMS cranial window implantation for 10 weeks (right column) versus an age-matched normal rat (left column). Images were captured from three different locations (a, b, c) at the cranial window. There were no clear differences in histological analysis of microglial

cells and astrocytes of animals with and without the PDMS chronic cranial window.

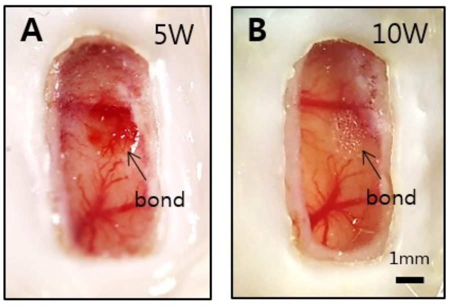

Supplementary Fig. S9 Treatment of a clear cyanoacrylate bond to the penetration points of PDMS. In penetration experiments, to avoid potential CSF leakage, a clear cyanoacrylate bond was applied immediately after the electrode was removed (A). After another 5 weeks (B), no signs of breakage of PDMS or CSF leakage were observed (B).

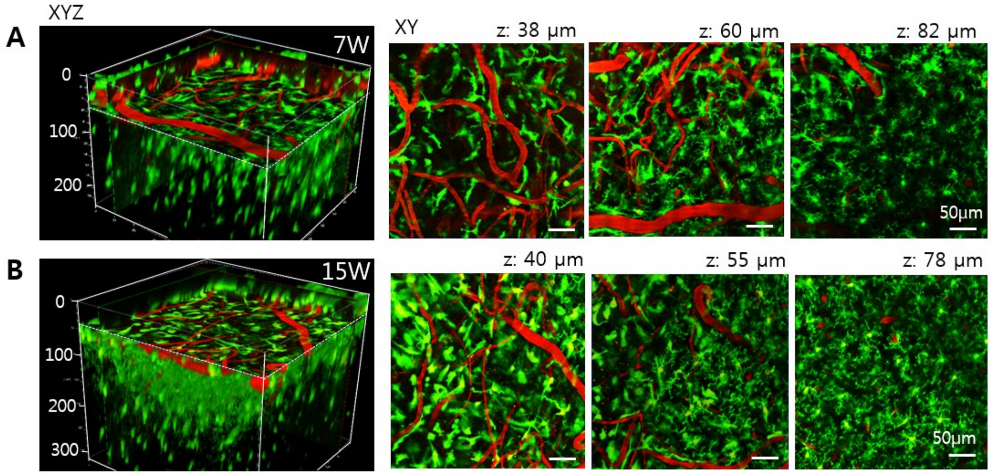

Supplementary Fig. S10 Two-photon microscopic images for vessels and microglial cells of a Cx3Cr1$^{GFP+/-}$ Tg mouse with a PDMS chronic cranial window. Vessels were visualized after injection of dextran-rhodamine (70000 molecular weight). XYZ 3D images were obtained from a mouse at 7 weeks (A) and 15 weeks (B) post-PDMS cranial window implantation surgery. XY-plane images are shown at three different depths.

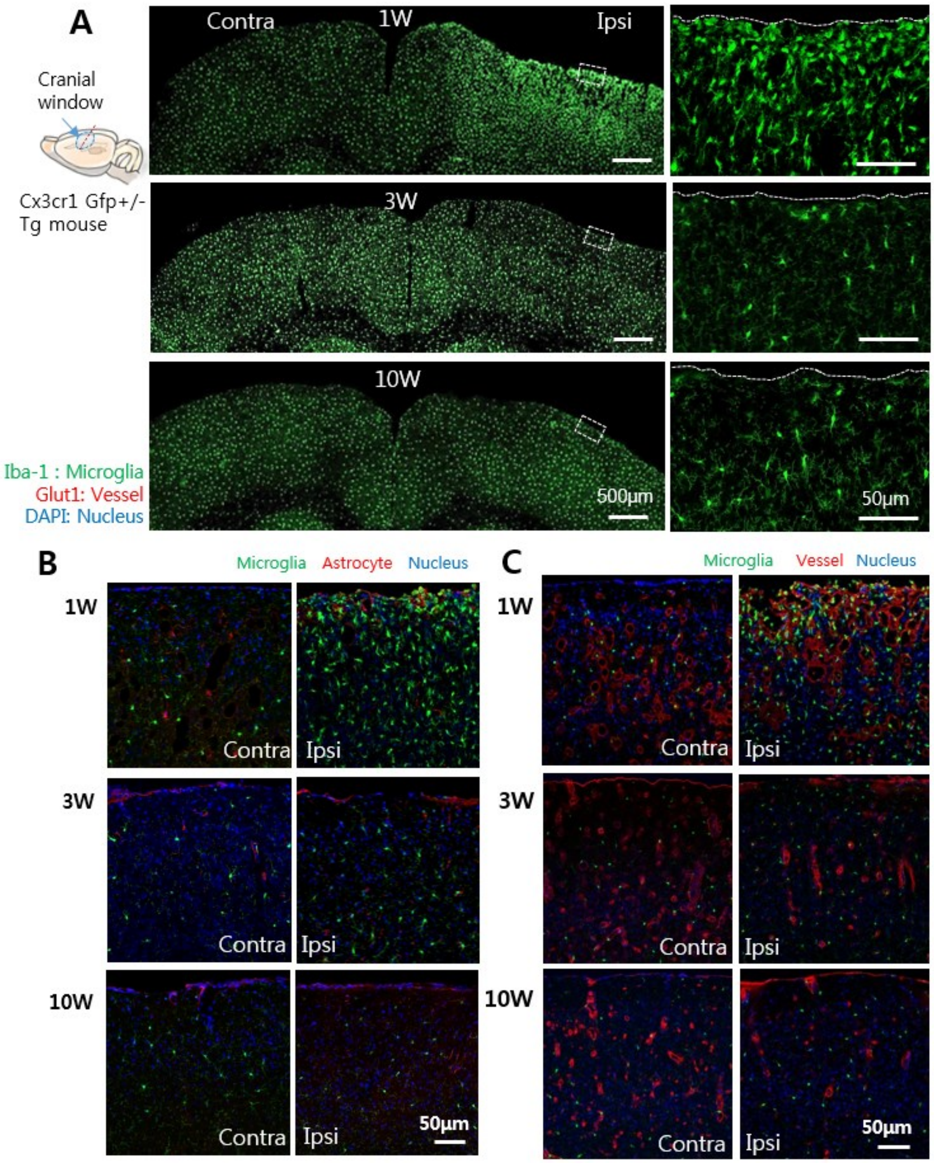

Supplementary Fig. S11 Immunohistochemical analysis of cortical tissue under the PDMS cranial window at 1, 3, and 10 weeks post-imaplantation surgery. (A) Cortical microglial cells in coronally sectioned Cx3Cr1$^{GFP+/-}$ Tg mouse brain slices were imaged by confocal microscope. Magnified images of the boxed sections in Ipsi are shown to the right. Other immunohistochemical study was examined for cortical vessels (Glut1), astrocytes (GFAP) and nuclei (DAPI) (B) or microglial cells (Iba1), astrocytes (GFAP) and nuclei (DAPI) (C). The images were captured from both contralateral and ipsilateral sides. Contra: Contralateral hemisphere; Ipsi: Ipsilateral hemisphere.

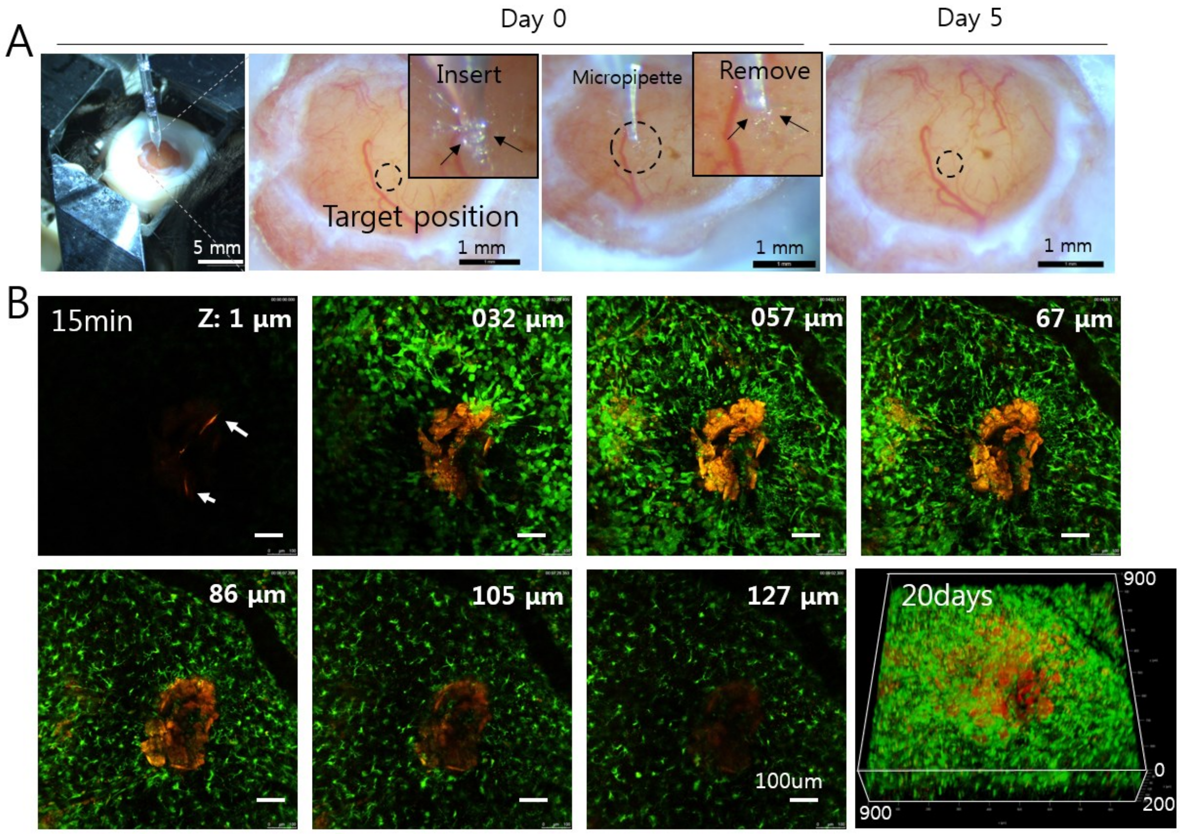

Supplementary Fig. S12 (A) The target position on PDMS window of the mouse was marked in a dotted circle. A glass micropipette filled with Cy3-labeled hydrogel dye is directly inserted into the target site and removed. Two black arrows represent the edge of the micropipette in the insert image at Day 0. After the removal of the micropipette, the injection site's edges are slightly noticeable under the surgical microscope with 60 x magnification but became invisible at 5 days post-injection. (B) The sign of injecting positon at the surface of the PDMS window can be found by the 2p confocal microscope (marked by two white arrows, at z-depth 1 µm). The Cy3-labeled hydrogel (red) is imaged in depth profile and distributed approximately from 30 to 130 µm in Z-depth in the cortical tissue. The Cy3-labeled hydrogel dye was presented in 3D volume image after 20 days post-injection.

Supplementary Videos (5)
Available in the seperate uploaded video files.

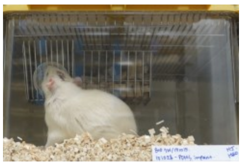

Supplementary Video S1. Video of rat behaviour at 7 weeks post-PDMS implantation. A single animal was housed in it's own home cage after cranial window implantation surgery. The animal exhibited normal grooming, drinking, feeding, and sleeping behavior.

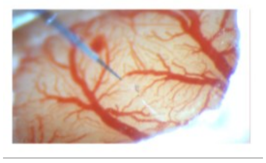

Supplementary Video S2. Direct insertion of a multi-channel electrode into the cortex through PDMS window. A soft electrode was inserted through a small slit made by an insulin syringe needle (sma ll needle size with 0.33 μm in diameter: 29 gauge) in the PDMS cranial window. The electrode was removed without creating any CSF leakage.

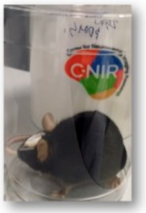

Supplementary Video S3. Video of mouse behaviour at 25 weeks post-PDMS implantation surgery.

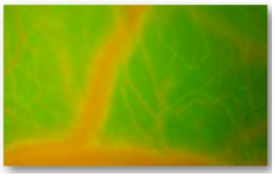

Supplementary Video S4. Video of the cortical blood flow after the administration of dextran-rhodamine into Cx3Cr1$^{GFP+/-}$ Tg mice. Video was captured using fluorescence (green) microscopy at 25 weeks post PDMS implantation surgery. The green highlighted spots on the cortical surface represent Cx3Cr1 GFP-positive microglia cells.

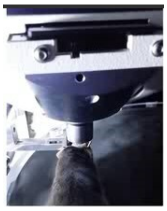

Supplementary Video S5. Locomotion video of an awake mouse during two-photon imaging.
